# A self-regulated expiratory flow device for mechanical ventilation: a bench study

**DOI:** 10.1186/s40635-024-00681-0

**Published:** 2024-10-16

**Authors:** Lianye Yang, Ubbo F. Wiersema, Shailesh Bihari, Roy Broughton, Andy Roberts, Nigel Kelley, Mark McEwen

**Affiliations:** 1 Biomedical Engineering Department, Flinders Medical Centre, South Adelaide Local Health Network, Adelaide, SA Australia; 2 Intensive and Critical Care Unit, Flinders Medical Centre, South Adelaide Local Health Network, Flinders Lane, Bedford Park, Adelaide, SA 5042 Australia; 3 College of Medicine and Public Health, Flinders University, Adelaide, Australia

**Keywords:** Energy dissipation, Expiratory resistive load, Flow-controlled expiration, Mandatory ventilation

## Abstract

**Introduction:**

Unregulated expiratory flow may contribute to ventilator-induced lung injury. The amount of energy dissipated into the lungs with tidal mechanical ventilation may be used to quantify potentially injurious ventilation. Previously reported devices for variable expiratory flow regulation (FLEX) require, either computer-controlled feedback, or an initial expiratory flow trigger. In this bench study we present a novel passive expiratory flow regulation device.

**Methods:**

The device was tested using a commercially available mechanical ventilator with a range of settings (tidal volume 420 ml and 630 ml, max. inspiratory flow rate 30 L/min and 50 L/min, respiratory rate 10 min^−1^, positive end-expiratory pressure 5 cmH_2_O), and a test lung with six different combinations of compliance and resistance settings. The effectiveness of the device was evaluated for reduction in peak expiratory flow, expiratory time, mean airway pressure, and the reduction of tidal dissipated energy (measured as the area within the airway pressure–volume loop).

**Results:**

Maximal and minimal reduction in peak expiratory flow was from 97.18 ± 0.41 L/min to 25.82 ± 0.07 L/min (*p* < 0.001), and from 44.11 ± 0.42 L/min to 26.30 ± 0.06 L/min, respectively. Maximal prolongation in expiratory time was recorded from 1.53 ± 0.06 s to 3.64 ± 0.21 s (*p* < 0.001). As a result of the extended expiration, the maximal decrease in I:E ratio was from 1:1.15 ± 0.03 to 1:2.45 ± 0.01 (*p* < 0.001). The greatest increase in mean airway pressure was from 10.04 ± 0.03 cmH_2_O to 17.33 ± 0.03 cmH_2_O. Dissipated energy was significantly reduced with the device under all test conditions (*p* < 0.001). The greatest reduction in dissipated energy was from 1.74 ± 0.00 J to 0.84 ± 0.00 J per breath. The least reduction in dissipated energy was from 0.30 ± 0.00 J to 0.16 ± 0.00 J per breath. The greatest and least percentage reduction in dissipated energy was 68% and 33%, respectively.

**Conclusions:**

The device bench tested in this study demonstrated a significant reduction in peak expiratory flow rate and dissipated energy, compared to ventilation with unregulated expiratory flow. Application of the device warrants further experimental and clinical evaluation.

**Supplementary Information:**

The online version contains supplementary material available at 10.1186/s40635-024-00681-0.

## Introduction

Ventilator-induced lung injury (VILI) has historically been attributed to delivery of excessive pressure (barotrauma), alveolar overdistension (volutrauma), and repetitive alveolar collapse and reopening (atelectotrauma) [1]. More recently, the contribution of flow and respiratory rate to VILI has been recognized [2]. This has led to the unifying concept of mechanical power, or ventilator delivered energy, as an integrated measure of the mechanical factors leading to VILI [2, 3]. The most injurious component of the delivered energy with each tidal breath is that part dissipated into the tissues [4]. The amount of dissipated energy may be measured as the area within the tidal airway pressure–volume loop [3, 5, 6]. Theoretically the amount of dissipated energy is minimized if flow is constant during both inspiration and expiration [7].

Although the flow characteristics of inspiration are readily controlled during mechanical ventilation, expiration is largely passive, driven by the elastic recoil of the lungs. Expiration usually manifests an exponential decaying flow waveform over time, with an abrupt initial high flow. Expiration may be particularly abrupt and injurious in patients with acute respiratory distress syndrome (ARDS) [8, 9]. In experimental lung injury, attenuation of the rapid initial flow during expiration, or application of constant expiratory flow, has been associated with reduced energy dissipation, reduced alveolar injury and improved ventilation of dorsal lung regions [6, 8, 10, 11]. Expiratory flow control may also decrease demand for positive end-expiratory pressure (PEEP), provide closer matching of inspiratory and expiratory compliance [10, 12], and reduce expiratory airway compression in patients with chronic obstructive pulmonary disease (COPD) [13].

Previous studies of flow-controlled expiration (FLEX) have utilized a variable resistor, adjusted by a computer-controlled linear motor. This requires continuous intratracheal pressure and a proprietary ventilator in flow-controlled ventilation mode (FCV) [14]. Recently, development of a mechanical, passive expiratory flow regulator was reported [15]. Although simple and effective the design requires an initial flow to trigger the device, which implies an unregulated high flow at the onset of expiration. This trigger threshold prevented the device from working effectively in 15% of healthy subjects [15].

In the present study, we report the development of a novel adjustable passive mechanical expiratory flow regulation FLEX device. The device was evaluated with a commercially available ventilator and test lung under a wide range of ventilator settings and test lung conditions.

## Methodology

**Fig. 1:**
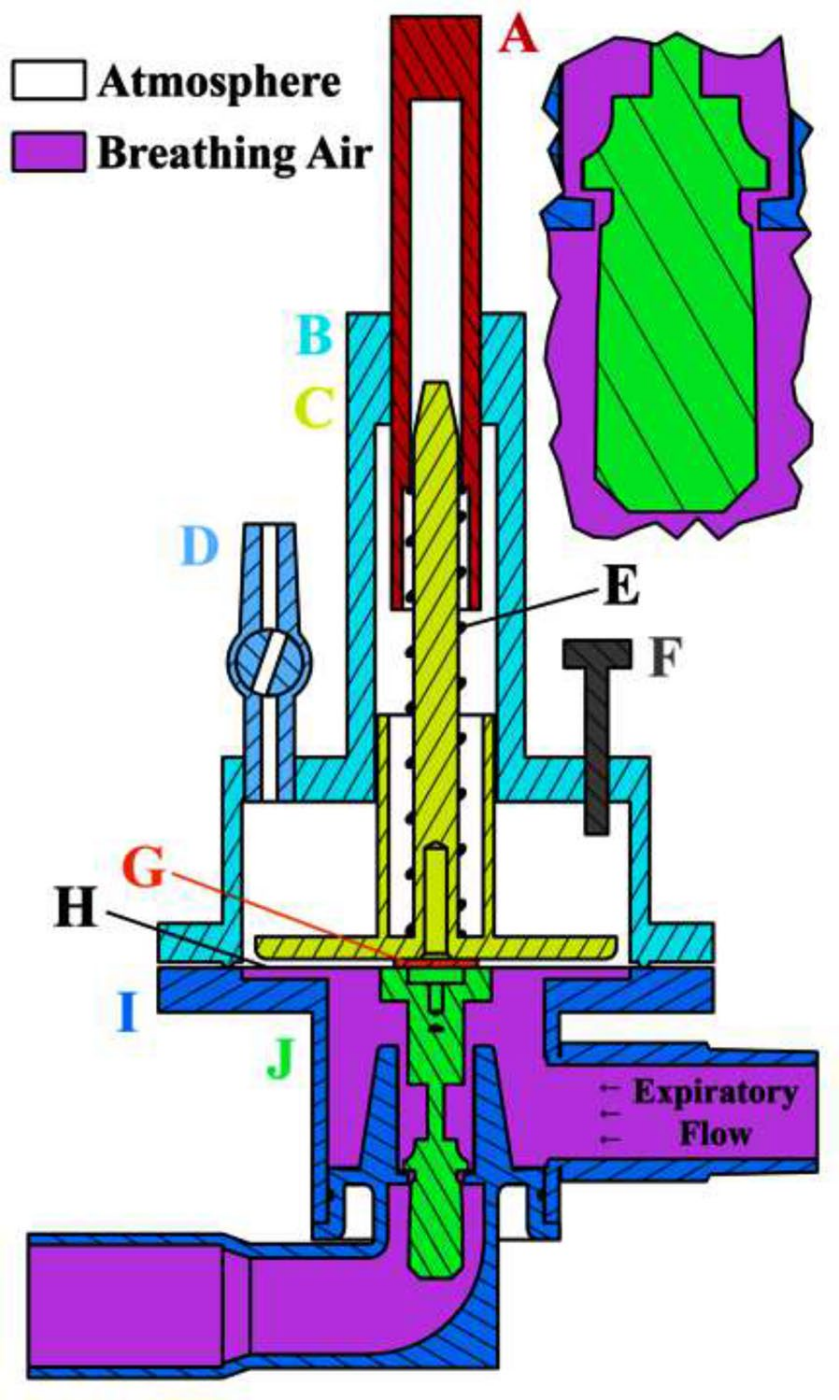
Cross section of the FLEX device. **A** Trigger pressure adjusting rod. **B** Damping chamber. **C** PEEP plate. **D** Damping vent-hole. **E** Adjustable spring. **F** PEEP plate range limiter. **G** Magnet. **H** Diaphragm. **I** Pilot Chamber. **J** Conical flow-control valve

The FLEX device consists of an inspiratory flow (one-way) check valve, a pilot chamber, a damping chamber, an adjustable spring-loaded PEEP plate, a diaphragm and a conical flow-control valve (Fig. 1). The diaphragm has a thickness of 0.1 mm and is made from highly elastic natural rubber. The diaphragm separates the pilot chamber from the damping chamber. The conical flow-control valve assembly is attached to the center of the diaphragm by a magnet.

The pilot chamber receives the inspiratory gas from the ventilator at the onset of inspiration. The inspiratory gas pressurizes the pilot chamber causing the diaphragm to lift the conical flow-control valve. This reduces the area of the expiratory orifice, because of the conical shape of the flow-control valve. To prevent occlusion, the flow-control valve is never fully closed. Later during expiration, the pressure in the pilot chamber decreases allowing the diaphragm to return to its starting position and the flow-control valve to progressively open, reducing the resistance to expiratory flow.

The damping chamber controls the rate at which the flow-control valve can rise and fall. The degree of damping is adjusted by altering the cross-sectional area of a vent hole (between 0 and 6.16 mm^2^) through which air in the damping chamber can be exhausted to atmosphere. Adjustments to the cross-sectional area of the vent hole were made mainly to damp out any sudden change in resistance or possible oscillation. The cross-sectional area was never set to zero.

The adjustable spring-loaded PEEP plate sits on top of the diaphragm. The PEEP plate counteracts the PEEP set on the ventilator. It also controls the minimum actuation pressure at which the flow-control valve is elevated and limits the maximum upward range of the flow-control valve (limiting the maximum expiratory resistance provided by the FLEX device).

The effectiveness of the FLEX device was evaluated with a test lung (Lung Simulator, Medshield, Essex, UK) using a range of different compliances and airways resistances. Compliance of the test lung was adjusted by applying a different number of springs to the bellows. The compliance profile of the test lung was determined using the super-syringe method under quasi-static conditions (50 ml increments with 2–3 s pause), up to a maximum lung volume of 800ml [16]. Resistance of the test lung was adjusted using a dial on the test lung and calibrated using measurements from the ventilator with an inspiratory pause. Resistance values between 10 and 16 $cm{H}_{2}O/(L\cdot {s}^{-1})$ were used. All tests were completed with a variation in airway resistance of ± 0.5 $cm{H}_{2}O/(L\cdot {s}^{-1})$.

The test lung was ventilated with a Puritan Bennett 980 Ventilator (Medtronic, Minneapolis, MN, USA) in volume control (constant inspiratory flow) mode with four sets of settings: 420 or 630 ml tidal volume (VT), and 30 or 50 L/min inspiratory flow. The respiratory rate was set to 10 breaths per minute and PEEP set to 5 cmH_2_O for all studies unless otherwise stated. No end-inspiratory pause was applied. Medical air, without supplemental oxygen was used.

To study the feasibility of the device at higher level of PEEP, four additional supplementary tests were conducted with PEEP level set to 15 cmH_2_O. The analyses of these supplementary tests at higher PEEP level were conducted separately to those tested at PEEP level of 5 cmH_2_O.

**Fig. 2:**
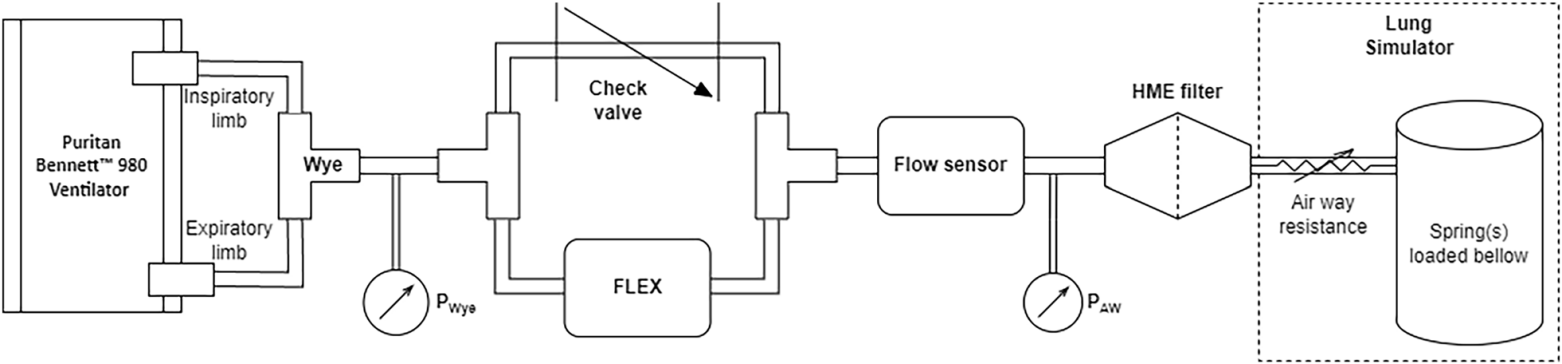
Block diagram of the experimental setup with the test lung. PWye: pressure at Wye between the FLEX device and the ventilator; PAW: airway pressure between the HME filter and flow sensor. HME filter: Heat and moisture exchange filter. The flow sensor was placed between the two pressure sensors, allowing resistance of the FLEX device to be calculated as as $\frac{{\text{P}}_{\text{AW}}-{\text{P}}_{\text{wye}}}{\text{Flow rate}}.$ For experiments conducted under baseline conditions the FLEX device was removed and the two ends of 22 mm diameter soft tubing were directly connected to complete the circuit; the inspiratory flow (one-way) check value always remained in the circuit

The experimental setup is illustrated in Fig. 2. A heat and moisture exchange filter was placed adjacent to the test lung and separated from the flow sensor (SFM3000, Sensirion, Stäfa, Switzerland) by a 20 cm long piece of rigid tubing with an inner diameter of 20.8 mm. The filter and rigid tubing were essential to condition the turbulent and jetting flow for accurate flow measurement by the flow sensor. Two pressure sensors (SM9541-100C-D-C-3-S, Carlsbad, CA, USA) were placed on either side of the flow sensor and FLEX device. The three sensors were sampled sequentially with a small latency between them, at a sampling rate of 500 Hz. For each test condition, 310 s of raw data were captured and converted to actual measurements in real time with a custom MATLAB application (Version R2023a, The MathWorks Inc., Natick, MA, USA).

Due to the additional circuit compliance of the experimental setup, the actual volume delivered to the test lung was less than the tidal volume set on the ventilator [17, 18]. The delivered tidal volume with the FLEX device in the experimental setup was also less than with the FLEX device removed. To compensate for this discrepancy, the inspired tidal volume was manually adjusted on the ventilator during each experiment to ensure matching of volumes between baseline and FLEX conditions. This ensured that comparable volumes were used for calculations of mechanical energy dissipation.

Post-processing and plotting of the recorded data was performed using a custom script written in MATLAB. No filtering was applied to the visualization of continuous data. However, a low-pass filter with pass-band frequency of 25 Hz, stop-band frequency of 80 Hz and stop-band attenuation of 40 dB was deployed to attenuate noise, prior to calculating benchmarking parameters and statistical analyses (see supplementary Fig. A). Continuous data from tidal breaths were ensemble averaged after alignment to the start of each inspiration. Volume was calculated by integration of flow over time. Inspiratory and expiratory phase energy per breath was calculated from integration of pressure and volume data according to Eq. 1, in which $P\left(V\right)$ is a function of pressure whose dependent is volume. The amount of energy dissipated per breath was computed as the difference between the inspiratory and expiratory energy per breath:

1 $$E = \,\int {P\left( V \right)\,\Delta V}$$

### Statistical analysis

Benchmarking parameters including peak expiratory flow, expiratory time, I:E ratio, mean airway pressure, inspiratory energy, and energy dissipation derived from each respiratory cycle were presented as mean ± standard deviation and are tested against normality with Anderson–Darling test. Significant differences between baseline and FLEX conditions were identified within each of the 24 bench tests (and each of the four supplementary tests), with paired-sample *T* tests if normally distributed or Wilcoxon’s signed rank test if not. Two-way analysis of variance (ANOVA) (on ranks when appropriate) was used to identify statistical significancy in energy dissipation considering the presence of a statistically significant difference in inspiratory energy. All statistical analyses were performed in MATLAB (Version R2023a, The MathWorks Inc., Natick, MA, USA). *P* < 0.01 was considered statistically significant.

## Results

**Fig. 3:**
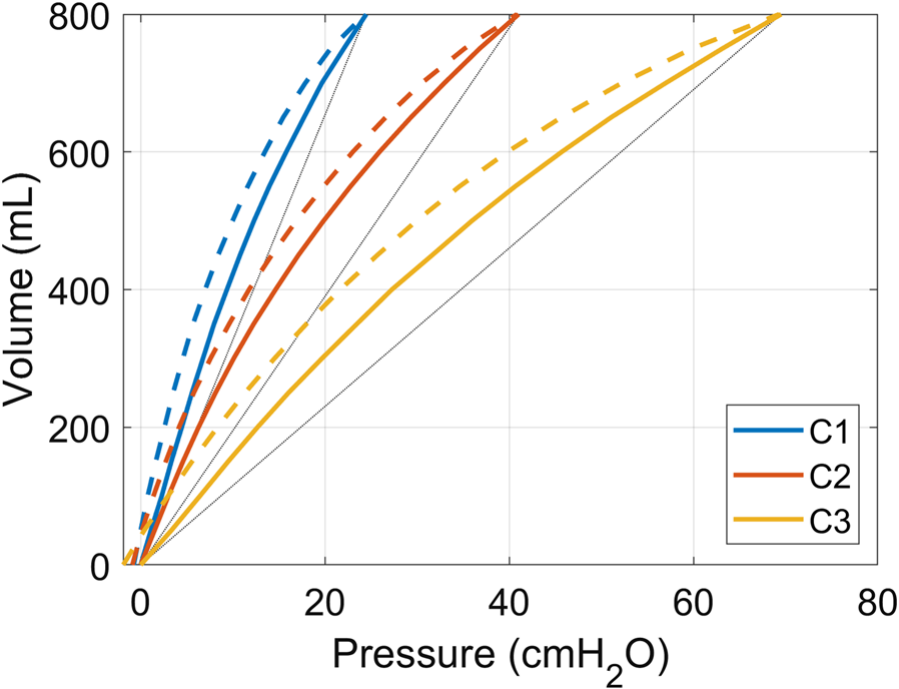
Quasi-static compliance profiles for the lung model characterised by the super-syringe technique. C1: Compliance profile 1, single spring-loaded bellow, chord compliance 32.68 ml/cmH_2_O. C2: Compliance profile 2, double spring-loaded bellow, chord compliance 19.50 ml/cmH_2_O. C3: Compliance profile 3, triple spring-loaded bellow, chord compliance 11.51 ml/cmH_2_O. Dash lines are linear approximation of the lung compliances

**Fig. 4:**
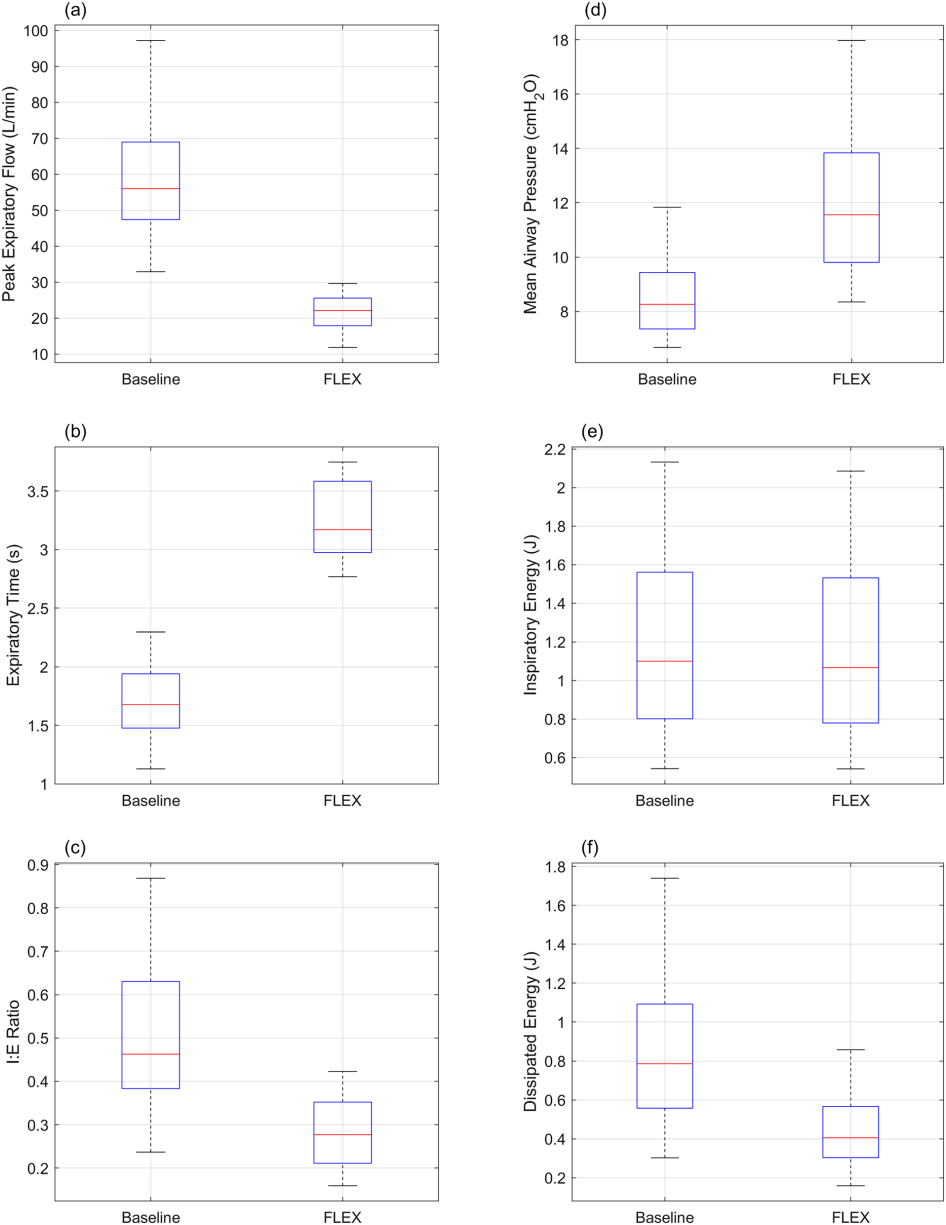
Box–Whisker summary of key performance parameters across all 24 bench tests. **a** Significant and unidirectional reduction is observed in peak expiratory flow with FLEX device in situ. **b** Significant and unidirectional increment is observed in expiratory time with FLEX device in situ. **c** Reduction is observed in I:E ratio with FLEX device in situ. **d** Elevation is observed in mean airway pressure with FLEX device in situ. **e** Insignificant difference is observed in inspiratory energy with/without FLEX device in situ. Small difference in inspiratory energy is favorable for the experiment. **f** Considerable reductions observed in dissipated energy with FLEX device in situ. For comprehensive test results and graphical representation of variations in key performance parameters, please refer to supplementary Table 1, Table 2, and Fig. B

Compliance characteristics of the test lung, as determined with the super-syringe technique, are shown in Fig. 3. In total 24 conditions were tested with and without the FLEX device. Comparisons across all 24 bench tests between baseline and FLEX conditions are summarized in Fig. 4. Key performance parameters related to flow are reported in Supplementary Table 1, and parameters related to pressure and energy are reported in Supplementary Table 2.

Peak expiratory flow was significantly reduced in all test cases with the FLEX device in situ (*p* < 0.001). The greatest reduction in expiratory flow was observed for test conditions where unregulated expiratory flow (no FLEX device in situ) was highest (from 97.18 ± 0.41 L/min to 25.82 ± 0.07 L/min). Minimal reduction in expiratory flow occurred where unregulated expiratory flow was low and when the duration of inspiration was longer. Nevertheless, the FLEX device was still able to reduce peak expiratory flow under such conditions (from 44.11 ± 0.42 L/min to 26.30 ± 0.06 L/min). A trend was observed across all 24 bench tests, in which the FLEX device facilitates a higher reducing of peak expiratory flow for ventilatory conditions with lower airway resistance and lung compliance at higher tidal volume. No obvious relationship between reduction in peak expiratory flow and inspiratory flow rate was found.

The duration of expiratory flow time was significantly prolonged in all test cases where the FLEX device was in situ (*P* < 0.001). The extent to which expiratory time was prolonged ranged between 1.53 ± 0.06 s unregulated to 3.64 ± 0.21 s with FLEX, and 2.25 ± 0.08 s unregulated to 3.17 ± 0.08 s with FLEX, at most and least, respectively. In general, across all 24 bench tests, the FLEX device was able to extend the expiratory time longer for ventilatory conditions with lower airway resistance, lung compliance, and tidal volume. No obvious relationship between prolonging in expiratory time and inspiratory flow rate was found.

The ratio of inspiration time to expiration time (I:E ratio) decreased significantly with the FLEX in situ in all test cases (*p* < 0.001). The maximal decrease in I:E ratio was from 1:1.15 ± 0.03 to 1:2.45 ± 0.01. The minimal decrease in I:E ratio was from 1:4.22 ± 0.01 to 1:6.28 ± 0.01. Across all 24 bench tests, greater decreases in I:E ratio were found in ventilatory conditions with lower airway resistance, lung compliance, higher tidal volume, and faster inspiratory flow rate.

Mean airway pressure was significantly elevated with the FLEX device in situ under all test conditions. The greatest increase in mean airway pressure was from 10.04 ± 0.03 cmH_2_O to 17.33 ± 0.03 cmH_2_O. The least increase in airway pressure was from 6.68 ± 0.03 cmH_2_O to 8.35 ± 0.02 cmH_2_O. Throughout all 24 bench tests, ventilatory conditions with lower lung compliance and higher tidal volume shown a sign of greater elevations in mean airway pressure with FLEX device in situ.

Even though inspired tidal volumes were matched manually between unregulated and FLEX regulated breaths under the same test lung conditions, with the intention to minimize the difference in inspiratory energy, there were statistically significant differences in inspiratory energy for all tests (*p* < 0.001). Importantly, however, the differences in inspiratory energy per breath were bidirectional and insignificant in value. The greatest decrease in inspiratory energy with addition of the FLEX device was from 0.93 ± 0.00 J to 0.84 ± 0.00 J, and greatest increase in inspiratory energy with addition of the FLEX device was from 1.28 ± 0.00 J to 1.29 ± 0.00 J per breath.

**Fig. 5:**
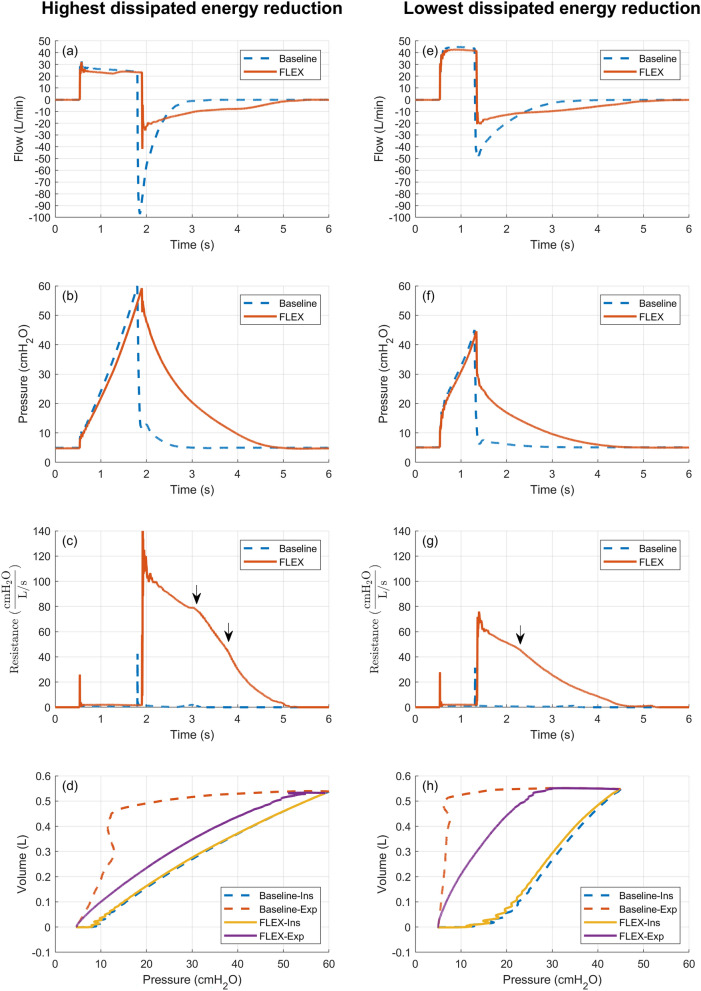
Two extreme performing cases of the FLEX device (percentagewise energy dissipation). Figures arranged on the left were results collected from the highest dissipated energy reduction case: Compliance profile C3, $airway resistance = 10 cm{H}_{2}O/(L\cdot {s}^{-1})$, ${V}_{T}=630 mL$, ${\dot{V}}_{MAX}=50L/min$. Figures arranged on the right were results collected from the lowest dissipated energy reduction case: Compliance profile C3, $airway resistance = 16 cm{H}_{2}O/(L\cdot {s}^{-1})$, ${V}_{T}=630 mL$, ${\dot{V}}_{MAX}=30L/min$. **a**, **e** illustrate the flow pattern for a single breath, with and without the FLEX device in situ. **b**, **f** illustrate the airway pressures for a single breath, with and without the FLEX device in situ. Peak pressures shown in the examples are slightly higher than the corresponding values under static (super syringe method) conditions, even when compensated for airway resistance. The higher pressures are due to the extra force required to overcome momentum and inflate the test lung. **c**, **g** illustrate the piecewise variable resistance profile of the FLEX device during expiration. Arrows indicate the turning points. The three turning points represent two types of physical changes. The first turning point at around 50 cmH_2_O/(L/s) in (**c**) and (**g**) is where the cross-sectional area of the flow-control valve changes more dramatically. This is also reflected in the transitioning region in the FLEX resistance vs. flow-control valve elevation curve (see supplementary Fig. F). The second turning point at around 80 cmH_2_O/(L/s), labelled only in (**c**), is a typical example when the maximum elevation of the flow-control valve is capped. In this case, the turning point indicates a moment when the pressure within the pilot chamber has reduced, and the flow-control valve starts to descend from its maximum elevation. These turning points were manually labelled. Note that the resistance induced by the flow sensor was negligible under baseline conditions and during inspiration. **d, h** illustrate the energy dissipation of a tidal breath, with and without the FLEX device in situ, as the area enclosed by the PV loop

Dissipated energy was significantly reduced with the FLEX device under all test conditions (*p* < 0.001). The greatest reduction in dissipated energy with the FLEX device was from 1.74 ± 0.00 J to 0.84 ± 0.00 J per breath. The least reduction in dissipated energy was from 0.30 ± 0.00 J to 0.16 ± 0.00 J per breath. The greatest percentage reduction in dissipated energy with the FLEX device was 68.2% (from 1.01 ± 0.00 J to 0.32 ± 0.00 J per breath). The least percentage reduction in dissipated energy was 33% (from 1.28 ± 0.01 J to 0.86 ± 0.00 J per breath) (see Fig. 5). Dissipated energy without FLEX device in situ was greater for ventilatory conditions with greater airway resistance, lower lung compliance, higher tidal volume and inspiratory flow rate. Therefore, absolute reductions in energy dissipation with FLEX device in situ were also more significant in conditions like such.

Observations consistent to the above summarized findings are evident in all four supplementary tests with PEEP level set to 15 cmH_2_O. Refer to supplementary Fig. C, Fig. D, Tables 1, and 2 for details.

Mechanical characterization of the Flex device with FLEX net driving pressure vs. flow-control valve elevation (Fig. E) and FLEX resistance vs. flow-control valve elevation (Fig. F) is provided in the supplement.

## Discussion

In this bench study, we have demonstrated the feasibility and efficacy of a novel pressure-driven adjustable expiratory flow-limiting device when used with a commercially available mechanical ventilator. A reduction in tidal (expiratory) dissipated energy was achieved under all test conditions over a wide range of test lung compliance and resistance.

Regulation of expiratory flow during mechanical ventilation has been demonstrated by other investigators using a (proprietary) computer regulated resistance, or a flow-driven regulator. Expiratory flow limitation may also be achieved with a simple fixed resistor. Our device has advantages over each of these other reported expiratory flow limiting methods. Compared to the proprietary flow control mode, our device is self-regulated, without the need for a computer-controlled resistance regulator [14]. Our device utilizes pressure driven flow regulation. Once the breathing system is pressurized during inspiration, our device is able to regulate flow as soon as expiration begins (Fig. 5). The previously reported passive flow-driven flow regulator requires a minimum expiratory flow rate to trigger expiratory flow limitation [15]. Our device is able to provide an approximately four times greater maximal resistance (at the onset of expiration) than the previously reported passive flow-driven flow regulator [15]. At the end of expiratory flow our device also provides a smooth transition to zero flow, rather than an abrupt change. Both flow control mode and the flow-driven flow regulator have, however, been more extensively evaluated [6, 8, 10, 11, 15, 19].

Expiratory flow regulation limits the abrupt initial peak flow at the onset of expiration, provides a higher mean airway pressure and reduces energy dissipation, compared to conventional mechanical ventilation with unregulated expiration [7, 20]. These features were all demonstrated in our study under all test conditions. Experimentally expiratory flow regulation has been shown to improve oxygenation, reduce alveolar injury and improve ventilation of dorsal lung regions in ARDS [8, 10, 11].

Damping of expiratory flow with a constant expiratory resistance device has been shown to reduce expiratory airway compression in patients with COPD and expiratory flow limitation [13, 21]. However, the constant resistance may predispose to dynamic hyperinflation and auto-PEEP [22]. Thus, a variable resistance is a necessary feature of any expiratory flow regulation device. Our design provides greater resistance to expiratory flow at the onset of expiration, with progressive decrease in resistance through the remainder of expiration. In our study, there was no significant increase in end-expiratory pressure (auto-PEEP) under any test conditions.

Theoretically minimum energy dissipation with tidal ventilation occurs with constant flow during both inspiration and expiration, and an I:E ratio of 1:1. This has been achieved with the proprietary flow control mode, where suction is applied when appropriate to ensure constant expiratory flow [7, 19, 23]. Constant expiratory flow is not possible with our device, and 1:1 ventilation is not easily achievable, whilst still ensuring that expiration is complete. However, we were still able to demonstrate a significant reduction in dissipated energy in all test cases. Furthermore, 1:1 ventilation is rarely applied in modern clinical practice.

## Limitations

The test lung used in our study consisted of a resistance (screw valve) and compliance (bellows) in series, without additional viscoelastic properties. Thus, energy dissipation was through the resistance only. The energy dissipation we observed is consistent with that found in other studies [12, 19].

Our FLEX device utilizes a stretchable diaphragm to control air flow. This modifies the circuit compliance, reducing the delivered tidal volume. The tidal volume setting of the ventilator thus had to be increased to compensate for this.

Addition of the FLEX device in the expiratory limb of the circuit interferes with ventilator pressure sensing during inspiration and expiration. To accurately measure airway pressures, a proximal pressure monitor between the patient airway and the device is required. Accurate measurement of energy dissipation within the patient airway and lungs would require proximal pressure and flow monitors to capture an accurate dynamic pressure–volume loop.

The FLEX device presented in this study is highly adjustable in its damping strength, minimum triggering pressure and maximal resistance range. In this study cross-sectional area of damping vent hole was manually adjusted to achieve the desired expiratory flow profile, based on experience. However, once set, the FLEX device is self-regulated. For clinical application, the additional length of tubing, used under the test conditions to improve accuracy of flow measurement, could be removed.

## Conclusion

The novel pressure-driven, adjustable FLEX device presented in this study was associated with a reduction in tidal energy dissipation under all bench study conditions tested. Future studies are required to determine the safety and efficacy of this device in an experimental or clinical setting.

## Supplementary Information

Supplementary material 1.

Supplementary material 2.

## Data Availability

The data sets used and/or analyzed during the current study are available from the corresponding author on reasonable request.

## Abbreviations

ANOVA: Analysis of variance
ARDS: Acute respiratory distress syndrome
COPD: Chronic obstructive pulmonary disease
FLEX: Flow-controlled expiration
PEEP: Positive end expiratory pressure
PV: Pressure volume
FCV: Flow-controlled ventilation
VILI: Ventilator-induced lung injury
$${V}_{T}$$: Tidal volume
${\dot{V}}_{MAX}$: Maximum inspiratory flow rate
RR: Respiratory rate
