# Supplementary material for: A self-regulated expiratory flow device for mechanical ventilation: a bench study
Source: Intensive Care Med Exp. 2024 Oct 16;12:92. doi: 10.1186/s40635-024-00681-0 (PMC11484996; doi:10.1186/s40635-024-00681-0)
Supplement: Supplementary file 1 — Supplementary material 1. [file 40635_2024_681_MOESM1_ESM.docx]

Supplementary: A self-regulated expiratory flow device for mechanical ventilation: a bench study

Lianye Yang^a^, Ubbo F Wiersema^b^, Shailesh Bihari^b,c,*^, Roy Broughton^a^, Andy Roberts^a^, Nigel Kelley^a^, Mark McEwen^a^

^a^Biomedical Engineering Department, Flinders Medical Centre, South Adelaide Local Health Network, Adelaide, SA, Australia.

^b^Intensive and Critical Care Unit, Flinders Medical Centre, South Adelaide Local Health Network, Adelaide, SA, Australia.

^c^College of Medicine and Public Health, Flinders University

^*^Corresponding author: Address - Department of ICU, Flinders Medical Centre, Flinders Lane, Bedford Park SA 5042. Email - Shailesh.Bihari@sa.gov.au, biharishailesh@gmail.com. Phone - + 61 8 82047288. Fax - +61 8 82045751.


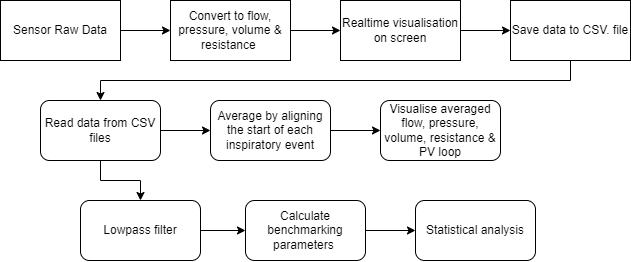


Fig. A. Data acquisition, processing, and analysis pipeline.


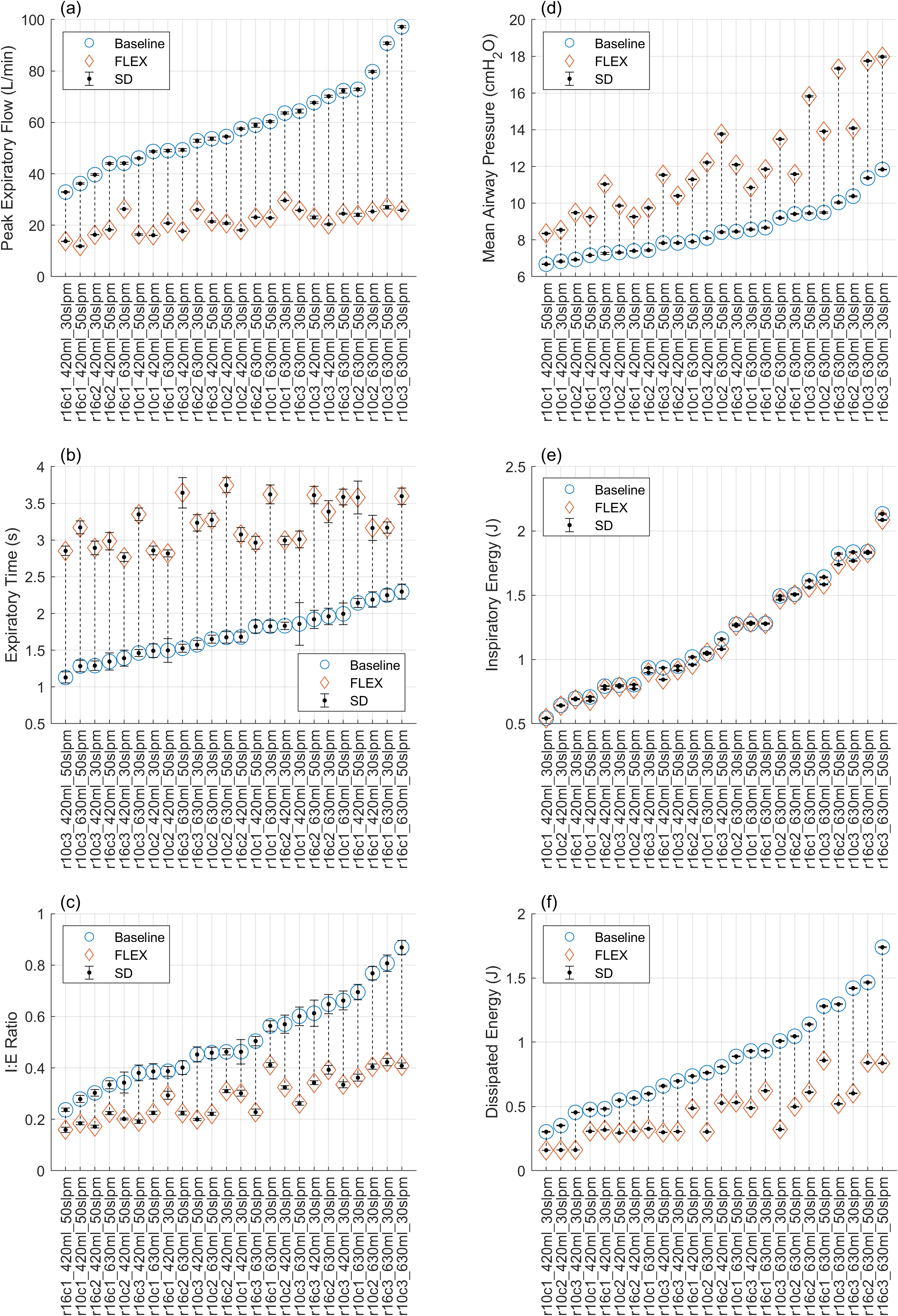


Fig. B. Examination results for 24 bench tests with PEEP set to 5 cmH_2_O. Test ID can be interpreted as r10 - $airway resistance = 10 cmH_{2}O/(L\cdot s^{-1})$; c1 - lung compliance profile c1; 420ml - $V_{T}=420 mL$; 30slpm - $\dot{V}_{MAX}=30 L/min$. Statistically significant differences between unregulated baseline and FLEX condition were identified within each test condition, using paired-sample T tests if data were normally distributed or Wilcoxon’s signed rank test if not. For energy dissipation, two-way ANOVA (on ranks when appropriate) were utilized to account for statistically significant differences in inspiratory energy. Results are presented by sorting baseline responses. (a): Statistically significant and unidirectional reductions observed in peak expiratory flow with FLEX device in situ (p < 0.001). (b): Statistically significant and unidirectional increments observed in expiratory time with FLEX device in situ (p < 0.001). (c): Statistically significant and unidirectional reductions observed in I:E ratio with FLEX device in situ (p < 0.001). (d): Statistically significant and unidirectional elevations observed in mean airway pressure with FLEX device in situ (p < 0.001). (e): Although statistically significant differences were observed in inspiratory energy with/without FLEX device in situ (p < 0.001), such differences were insignificant in value and are bidirectional. These differences in inspiratory energy are nowhere near the differences in dissipated energy. (f): Statistically significant and unidirectional reductions observed in dissipated energy with FLEX device in situ (p < 0.001).


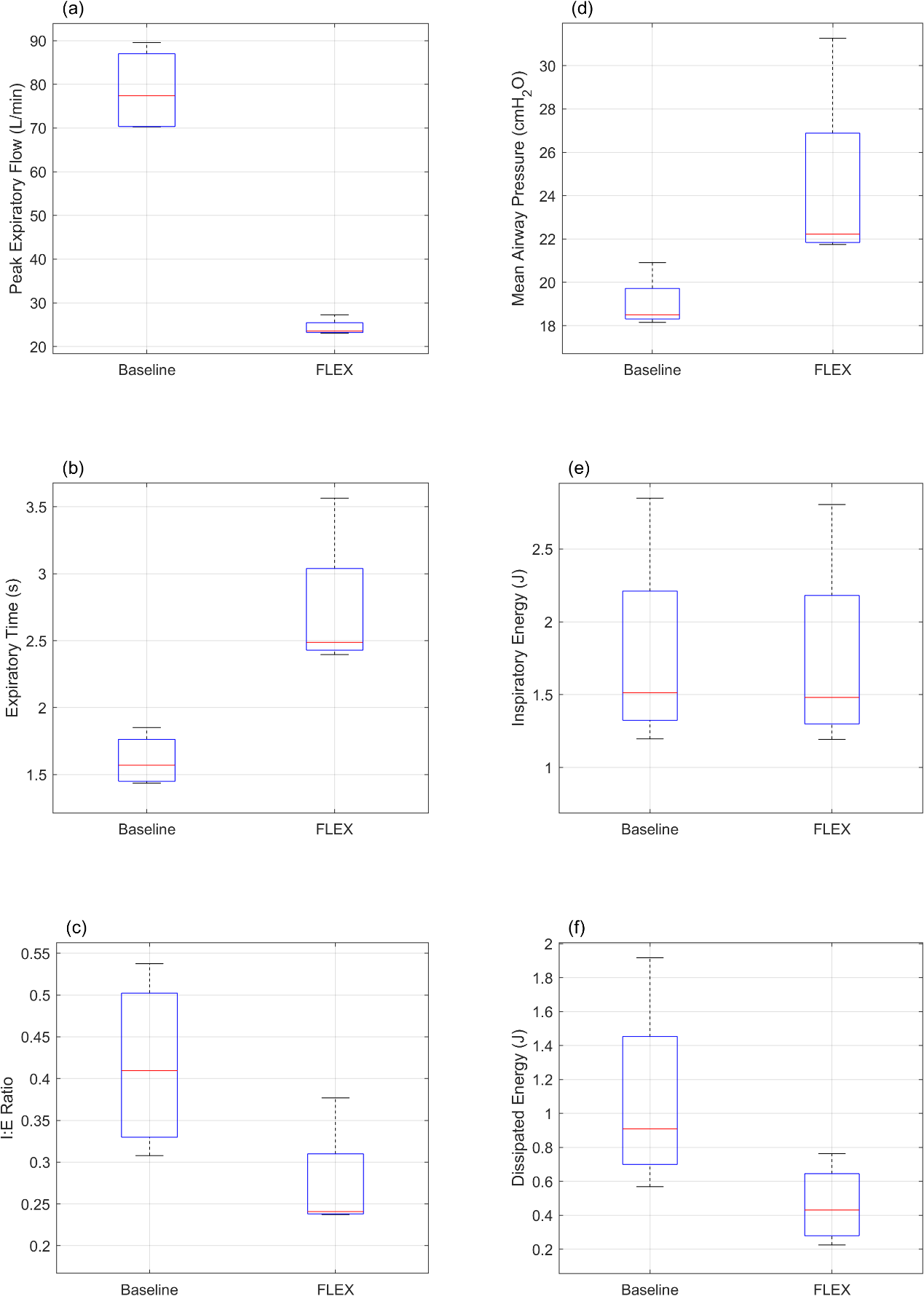


Fig. C. Box Whisker summary of key performance parameters across four supplementary tests with PEEP set to 15 cmH_2_O. (a): Significant and unidirectional reduction is observed in peak expiratory flow with FLEX device in situ. (b): Significant and unidirectional increment is observed in expiratory time with FLEX device in situ. (c): Reduction is observed in I:E ratio with FLEX device in situ. (d): Significant and unidirectional elevations are observed in mean airway pressure with FLEX device in situ. (e): Insignificant difference is observed in inspiratory energy with/without FLEX device in situ. Small difference in inspiratory energy is favorable for the experiment. (f): Considerable reduction observed in dissipated energy with FLEX device in situ.


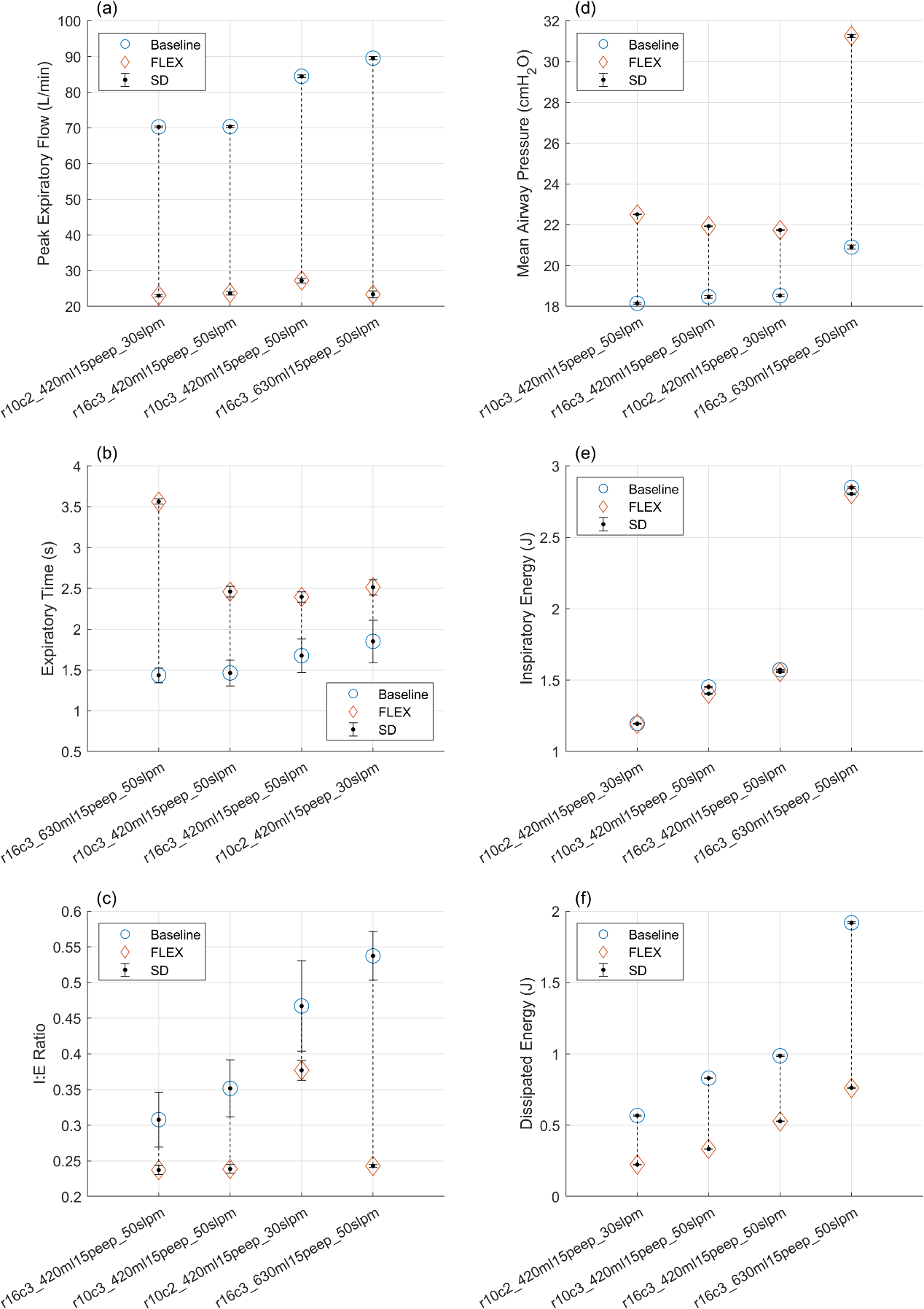


Fig. D. Examination results for 24 bench tests with PEEP set to 15 cmH_2_O. Results for the four supplementary tests presented in this figure can be interpreted the same way as per described in Fig. A.

Table 1: Examination of flow parameters. Test ID can be interpreted as r10 - $airway resistance = 10 cmH_{2}O/(L\cdot s^{-1})$; c1 - lung compliance profile c1; 420ml - $V_{T}=420 mL$; 30slpm - $\dot{V}_{MAX}=30 L/min$. Unless explicitly stated in the test ID, $PEEP=5 cmH_{2}O$. b: indicates the best performing case presented in Fig 5. w: indicates the worst performing case presented in Fig 5. *: Statistically significant differences between unregulated baseline and FLEX condition were identified within each test condition, using paired-sample T tests if data were normally distributed or Wilcoxon’s signed rank test if not.

| **Test ID** | **Peak expiratory flow (L/min) p < 0.001^*^** | | **Expiratory Time (s) p < 0.001^*^** | | **I:E ratio p < 0.001^*^** | |
| --- | --- | --- | --- | --- | --- | --- |
|  | **Unregulated** | **FLEX** | **Unregulated** | **FLEX** | **Unregulated** | **FLEX** |
| r10c1_420ml_30slpm | 48.69±0.31 | 16.09±0.06 | 1.86±0.29 | 3.01±0.11 | 1:2.16±0.048 | 1:3.32±0.011 |
| r10c1_420ml_50slpm | 46.11±0.21 | 16.38±0.22 | 1.82±0.09 | 2.96±0.09 | 1:3.59±0.014 | 1:5.42±0.006 |
| r10c1_630ml_30slpm | 63.61±0.43 | 29.62±0.08 | 1.83±0.08 | 3.62±0.13 | 1:1.44±0.03 | 1:2.76±0.014 |
| r10c1_630ml_50slpm | 60.36±0.36 | 22.81±0.07 | 2±0.15 | 3.59±0.11 | 1:2.59±0.03 | 1:4.44±0.007 |
| r10c2_420ml_30slpm | 57.53±0.31 | 18.08±0.24 | 1.49±0.09 | 2.86±0.06 | 1:1.76±0.035 | 1:3.08±0.007 |
| r10c2_420ml_50slpm | 54.49±0.26 | 20.74±0.25 | 1.5±0.16 | 2.82±0.05 | 1:2.92±0.041 | 1:4.97±0.003 |
| r10c2_630ml_30slpm | 79.73±0.35 | 25.35±0.04 | 1.65±0.05 | 3.27±0.09 | 1:1.3±0.025 | 1:2.47±0.01 |
| r10c2_630ml_50slpm | 72.87±0.47 | 23.99±0.61 | 1.68±0.08 | 3.75±0.1 | 1:2.18±0.022 | 1:4.53±0.006 |
| r10c3_420ml_30slpm | 70.18±0.35 | 20.34±0.21 | 1.29±0.06 | 2.89±0.09 | 1:1.51±0.036 | 1:2.99±0.011 |
| r10c3_420ml_50slpm | 67.68±0.37 | 22.99±0.53 | 1.13±0.08 | 2.85±0.06 | 1:2.21±0.029 | 1:5.02±0.004 |
| r10c3_630ml_30slpm^b^ | 97.18±0.41 | 25.82±0.07 | 1.46±0.05 | 3.35±0.08 | 1:1.15±0.028 | 1:2.45±0.01 |
| r10c3_630ml_50slpm | 90.8±0.46 | 26.96±0.67 | 1.28±0.08 | 3.17±0.09 | 1:1.66±0.036 | 1:3.83±0.007 |
| r16c1_420ml_30slpm | 32.89±0.20 | 13.77±0.05 | 2.19±0.10 | 3.16±0.17 | 1:2.58±0.019 | 1:3.41±0.016 |
| r16c1_420ml_50slpm | 36.21±0.23 | 11.84±0.22 | 2.14±0.06 | 3.58±0.22 | 1:4.22±0.007 | 1:6.28±0.01 |
| r16c1_630ml_30slpm | 44.11±0.42 | 26.3±0.06 | 2.25±0.08 | 3.17±0.08 | 1:1.78±0.02 | 1:2.43±0.009 |
| r16c1_630ml_50slpm^w^ | 48.99±0.49 | 20.76±0.04 | 2.3±0.10 | 3.6±0.11 | 1:2.99±0.015 | 1:4.46±0.006 |
| r16c2_420ml_30slpm | 39.66±0.31 | 16.28±0.05 | 1.83±0.05 | 3±0.06 | 1:2.16±0.013 | 1:3.23±0.006 |
| r16c2_420ml_50slpm | 44.01±0.38 | 18.2±0.03 | 1.68±0.07 | 3.08±0.09 | 1:3.3±0.013 | 1:5.83±0.005 |
| r16c2_630ml_30slpm | 52.9±0.61 | 26.01±0.07 | 1.96±0.11 | 3.39±0.15 | 1:1.54±0.038 | 1:2.55±0.017 |
| r16c2_630ml_50slpm | 58.9±0.76 | 23.06±0.05 | 1.92±0.12 | 3.61±0.12 | 1:2.49±0.026 | 1:4.47±0.007 |
| r16c3_420ml_30slpm | 49.29±0.49 | 17.68±0.03 | 1.39±0.11 | 2.77±0.06 | 1:1.63±0.05 | 1:2.92±0.007 |
| r16c3_420ml_50slpm | 53.65±0.65 | 21.39±0.24 | 1.35±0.12 | 2.99±0.12 | 1:2.63±0.031 | 1:5.25±0.007 |
| r16c3_630ml_30slpm | 64.41±0.71 | 25.77±0.07 | 1.57±0.06 | 3.24±0.11 | 1:1.24±0.032 | 1:2.37±0.014 |
| r16c3_630ml_50slpm | 72.32±0.9 | 24.49±0.2 | 1.53±0.06 | 3.64±0.21 | 1:1.98±0.019 | 1:4.39±0.012 |
| r10c2_420ml15peep_30slpm | 70.29±0.25 | 23.03±0.32 | 1.85±0.26 | 2.51±0.09 | 1:2.14±0.063 | 1:2.65±0.014 |
| r10c3_420ml15peep_50slpm | 84.47±0.38 | 27.23±0.61 | 1.46±0.16 | 2.46±0.06 | 1:2.84±0.040 | 1:4.19±0.006 |
| r16c3_420ml15peep_50slpm | 70.39±0.28 | 23.67±0.46 | 1.68±0.21 | 2.4±0.06 | 1:3.25±0.040 | 1:4.21±0.006 |
| r16c3_630ml15peep_50slpm | 89.56±0.44 | 23.38±0.95 | 1.43±0.09 | 3.57±0.03 | 1:1.86±0.034 | 1:4.12±0.002 |

Table 2: Examination of pressure and energy parameters. Test ID can be interpreted as r10 - $airway resistance = 10 cmH_{2}O/(L\cdot s^{-1})$; c1 - lung compliance profile c1; 420ml - $V_{T}=420 mL$; 30slpm - $\dot{V}_{MAX}=30 L/min$. Unless explicitly stated in the test ID, $PEEP=5 cmH_{2}O$. b: indicates the best performing case presented in Fig 5. w: indicates the worst performing case presented in Fig 5. *: Statistically significant differences between unregulated baseline and FLEX condition were identified within each test condition, using paired-sample T tests if data were normally distributed or Wilcoxon’s signed rank test if not. #: Statistically significant differences between unregulated baseline and FLEX condition were identified within each test condition, using two-way ANOVA (on ranks when appropriate) to account for statistically significant differences in inspiratory energy.

| **Test ID** | **Mean pressure (cm H_2_O) p < 0.001^*^** | | **Inspiratory energy (J) p < 0.001^*^** | | **Energy dissipation (J) p < 0.001^#^** | |
| --- | --- | --- | --- | --- | --- | --- |
|  | **Baseline** | **FLEX** | **Baseline** | **FLEX** | **Baseline** | **FLEX** |
| r10c1_420ml_30slpm | 6.83±0.03 | 8.55±0.02 | 0.542±0.002 | 0.541±0.002 | 0.303±0.002 | 0.159±0.0004 |
| r10c1_420ml_50slpm | 6.68±0.03 | 8.35±0.02 | 0.707±0.003 | 0.678±0.002 | 0.477±0.002 | 0.307±0.001 |
| r10c1_630ml_30slpm | 8.57±0.03 | 10.85±0.01 | 1.043±0.003 | 1.053±0.002 | 0.6±0.003 | 0.326±0.001 |
| r10c1_630ml_50slpm | 7.91±0.01 | 11.3±0.01 | 1.276±0.004 | 1.286±0.003 | 0.889±0.003 | 0.531±0.001 |
| r10c2_420ml_30slpm | 7.31±0.04 | 9.87±0.03 | 0.643±0.003 | 0.64±0.002 | 0.352±0.002 | 0.161±0.001 |
| r10c2_420ml_50slpm | 6.93±0.02 | 9.48±0.01 | 0.803±0.003 | 0.771±0.002 | 0.549±0.002 | 0.294±0.001 |
| r10c2_630ml_30slpm | 9.49±0.04 | 13.91±0.01 | 1.273±0.004 | 1.261±0.002 | 0.763±0.003 | 0.302±0.001 |
| r10c2_630ml_50slpm | 8.42±0.04 | 13.77±0.04 | 1.494±0.006 | 1.465±0.004 | 1.046±0.005 | 0.498±0.002 |
| r10c3_420ml_30slpm | 8.1±0.02 | 12.21±0.03 | 0.8±0.002 | 0.788±0.002 | 0.454±0.001 | 0.162±0.001 |
| r10c3_420ml_50slpm | 7.26±0.06 | 11.04±0.03 | 0.948±0.004 | 0.916±0.003 | 0.66±0.005 | 0.299±0.001 |
| r10c3_630ml_30slpm^b^ | 11.37±0.02 | 17.75±0.02 | 1.641±0.003 | 1.582±0.003 | 1.01±0.002 | 0.321±0.001 |
| r10c3_630ml_50slpm | 9.45±0.03 | 15.82±0.04 | 1.835±0.005 | 1.767±0.005 | 1.295±0.004 | 0.52±0.002 |
| r16c1_420ml_30slpm | 7.4±0.01 | 9.26±0.03 | 0.696±0.002 | 0.689±0.002 | 0.482±0.001 | 0.318±0.001 |
| r16c1_420ml_50slpm | 7.16±0.004 | 9.26±0.02 | 0.934±0.002 | 0.843±0.002 | 0.737±0.001 | 0.486±0.001 |
| r16c1_630ml_30slpm | 9.41±0.02 | 11.58±0.01 | 1.279±0.004 | 1.276±0.002 | 0.933±0.003 | 0.622±0.001 |
| r16c1_630ml_50slpm^w^ | 8.66±0.02 | 11.85±0.02 | 1.614±0.005 | 1.559±0.003 | 1.281±0.005 | 0.858±0.002 |
| r16c2_420ml_30slpm | 7.83±0.01 | 10.4±0.02 | 0.792±0.002 | 0.77±0.001 | 0.566±0.001 | 0.309±0.0005 |
| r16c2_420ml_50slpm | 7.44±0.03 | 9.75±0.02 | 1.019±0.003 | 0.957±0.003 | 0.809±0.002 | 0.526±0.001 |
| r16c2_630ml_30slpm | 10.38±0.03 | 14.08±0.03 | 1.507±0.004 | 1.503±0.003 | 1.139±0.003 | 0.61±0.002 |
| r16c2_630ml_50slpm | 9.2±0.03 | 13.49±0.02 | 1.821±0.005 | 1.738±0.004 | 1.465±0.004 | 0.84±0.002 |
| r16c3_420ml_30slpm | 8.45±0.02 | 12.1±0.02 | 0.934±0.002 | 0.897±0.001 | 0.698±0.002 | 0.305±0.001 |
| r16c3_420ml_50slpm | 7.83±0.02 | 11.54±0.02 | 1.158±0.003 | 1.08±0.003 | 0.932±0.002 | 0.488±0.001 |
| r16c3_630ml_30slpm | 11.83±0.02 | 17.97±0.04 | 1.837±0.003 | 1.828±0.003 | 1.421±0.002 | 0.602±0.002 |
| r16c3_630ml_50slpm | 10.04±0.03 | 17.33±0.03 | 2.132±0.005 | 2.085±0.004 | 1.739±0.004 | 0.836±0.002 |
| r10c2_420ml15peep_30slpm | 18.53±0.05 | 21.74±0.04 | 1.2±0.003 | 1.19±0.002 | 0.57±0.003 | 0.22±0.001 |
| r10c3_420ml15peep_50slpm | 18.15±0.04 | 22.51±0.02 | 1.45±0.003 | 1.41±0.003 | 0.83±0.002 | 0.33±0.002 |
| r16c3_420ml15peep_50slpm | 18.47±0.06 | 21.93±0.02 | 1.57±0.004 | 1.56±0.003 | 0.99±0.004 | 0.53±0.001 |
| r16c3_630ml15peep_50slpm | 20.91±0.1 | 31.26±0.06 | 2.85±0.006 | 2.8±0.006 | 1.92±0.007 | 0.76±0.003 |

**Flex device mechanical characterisation:** The FLEX device’s characteristics were examined in a two-step approach. In the first step, the expiratory outlet was occluded, and a constant pressure was applied to the pilot chamber with a syringe. Due to the pressure difference between the pilot chamber and damping chamber, the diaphragm was inflated, and the flow-control valve was elevated. The elevation of the flow-control valve was increased in 1mm steps from 0mm to 18mm. The static pressure, or “net driving pressure”, required to elevate the flow-control valve to a certain height, was measured and presented in Fig. E.

In application setting, this net driving pressure can be expressed as flow:

$$P_{net}=P_{awy}-P_{\bar{peep}}-P_{weight}-P_{spring}$$

In which $P_{net}$ is the net driving pressure to actuate the diaphragm. $P_{awy}$ is the pressure within the pilot chamber, or airway pressure. $P_{\bar{peep}}$ represents the PEEP pressure that was compensated by the PEEP adjusting spring. $P_{weight}$ represents the gravity of the PEEP adjusting plate assembly acting on the diaphragm which is a constant. $P_{spring}$ represents the additional spring force acting on the diaphragm as the diaphragm move upwards to compress the spring, which is linear to the displacement of the diaphragm.

Assuming $P_{\bar{peep}}$ always equal PEEP. The above equation can be rearranged as:

$$P_{net}+ P_{weight}+P_{spring} =P_{awy}-P_{peep}$$

Since $P_{net}$ can be expressed as an equation of flow-control valve displacement, same as $P_{spring}$, and $P_{weight}$ is a constant. These three terms can be combined into a compensated net driving pressure noted as $P_{\bar{net}}$.

The PEEP adjusting plate assembly weights 38.4066g. The diaphragm has an area of approx. 0.00384 m^2^. Hence the constant pressure applied on the diaphragm when the FLEX device is in the up-right position is approx. 0.997303 cmH_2_O.

The adjusting spring has a spring constant of 0.08 N/mm. By applying Hooke’s law, the $P_{spring}$ can be expressed as:

$$P_{spring}=\frac{0.08N/{mm}\times\Delta d}{0.00384 m^{2}}\times0.0101972{cmH_{2}O}/{Pa}$$

By adding the $P_{weight}$ and $P_{spring}$ terms to the $P_{net}$, a new relationship between $P_{awy}-P_{peep}$ and displacement of the flow-control valve can be derived.


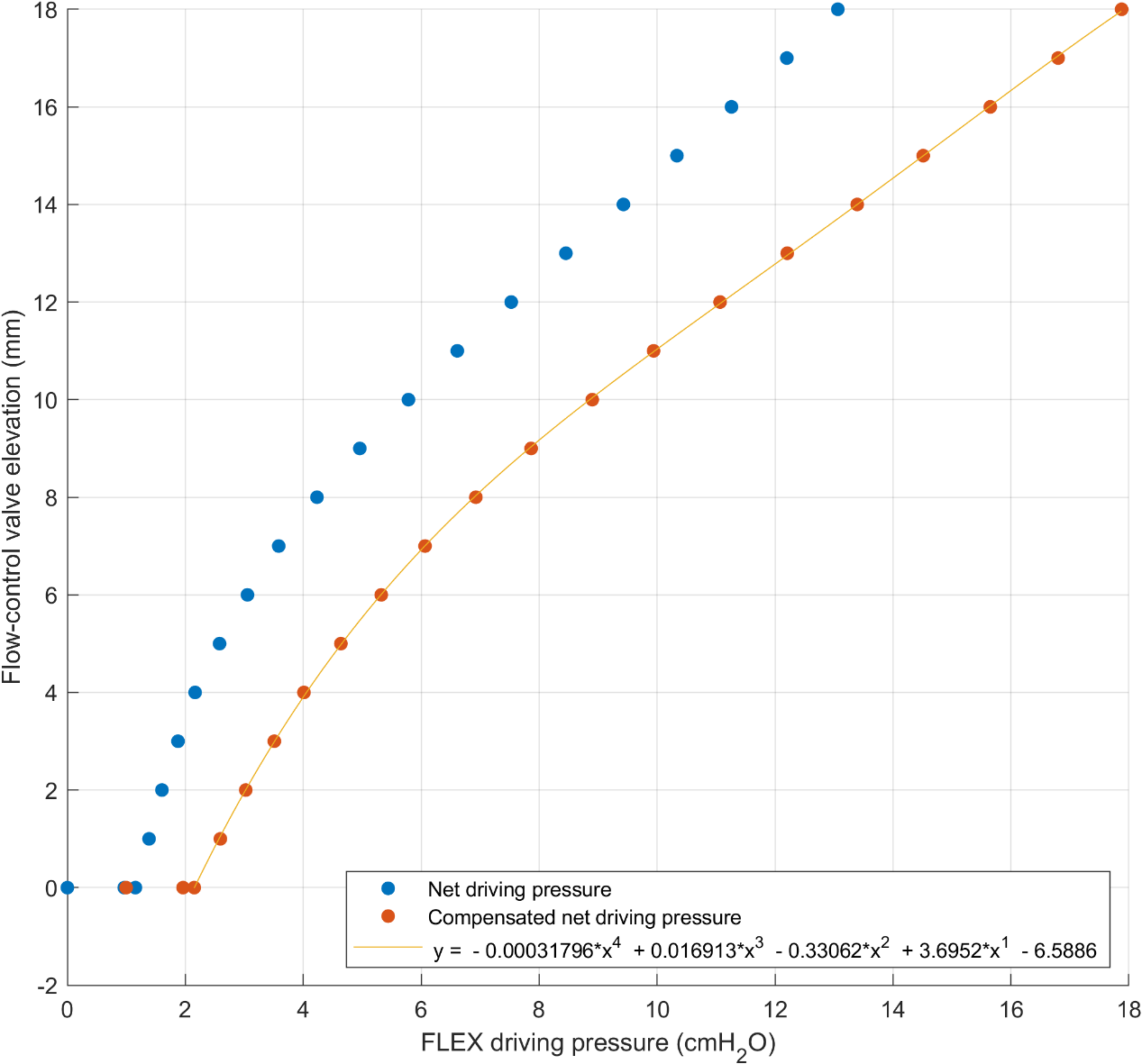


Fig. E. FLEX device flow-control valve elevation at different net driving pressure. The blue dots represent experimentally measured pressures within maximum standard deviation of 0.08. Red dots represent derived pressures when the downwards pressure of the spring and PEEP plate were considered. Yellow line is a 4th order approximation of the relationship between flow-control valve elevation and compensated net driving pressure.

The second step was to quantify resistance introduced by the FLEX device when the flow-control valve is elevated by a net driving pressure to a certain height. A fan was used to generate a constant net driving pressure with air flow through the FLEX device.

The net driving pressure was tuned, by changing the fan speed, until it equalized with the pressure required to elevate the flow-control valve to the target height (i.e. 1mm, 2mm). The air flow through the FLEX device and the pressure drop was measured to calculate resistance of the FLEX device. A 500Hz sampling rate with a two-second averaging window was used to produce one resistance readout. Ten randomly chosen non-overlapping resistance readings were averaged and standard deviations were derived. Results are presented in Fig. F.


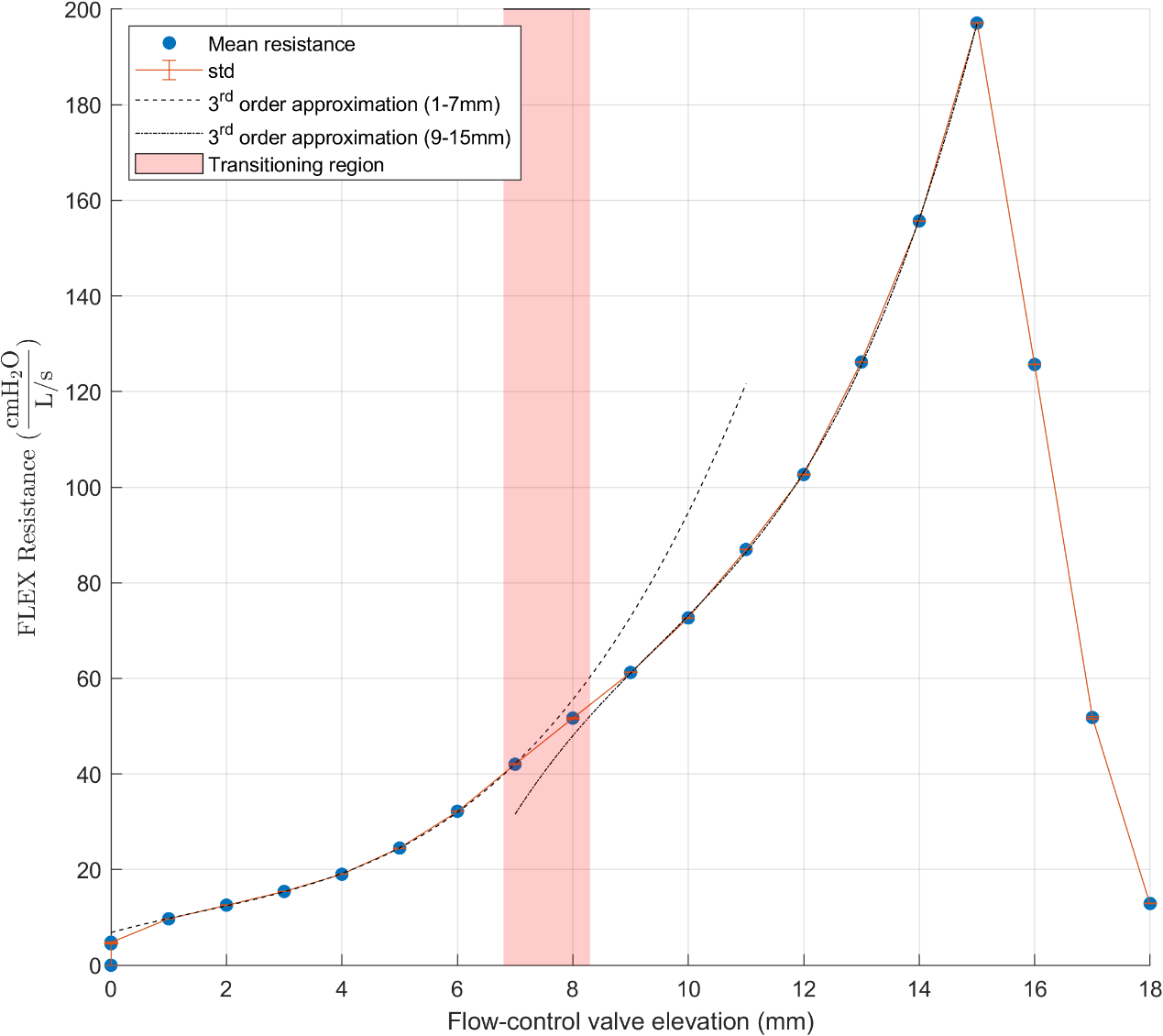


Fig. F. FLEX device resistance at certain flow-control valve elevation driven by static pressure. Refer to Fig. E. for static driving pressure required to elevate the flow-control valve to a certain height. Blue dots are the averaged resistance of the FLEX device. Maximum standard deviation was less than 0.21. Two piecewise 3rd order approximations of the resistance and flow-control valve elevation relationships were presented. Piecewise approximations were used because the physical shape of the flow-control valve elevation changes more dramatically in the transitioning region highlighted in red. Flow-control valve elevation greater than 15mm is beyond the range of normal operation.

Although the two profiles can be combined and the relationship between driving pressure and FLEX resistance can be derived, this can be misleading.

Travel of the flow-control valve assembly can be limited by the range limiter, for example at 10mm. When a driving pressure higher than 9 cmH_2_O is applied, the resistance of the FLEX will be higher than that indicated in Fig. F. Therefore, the pressure vs. resistance profile cannot be described with the same relationship when the flow-control valve is capped at a maximum height.

The above relationships between flow-control valve elevation, FLEX resistance, and FLEX driving pressure are only applicable when the flow-control valve starts to descend as the driving pressure drops below the pressure required to hold the flow-control valve at the capped position.

In addition, the above relationship was derived under static conditions, and system dynamics such as the damping effect were not accounted for. For a typical respiratory cycle, the pilot chamber is pressurised during inspiration, the damping chamber would also be pressurised because the damping effect. Pressure within the damping chamber will be positive. This positive pressure within the damping chamber reduces the net driving pressure. Hence FLEX resistance would be lower than with the static model in the early phase of the expiration.

As airway pressure reduces and the diaphragm starts to move downwards, the pressure within the damping chamber would start moving towards negative. Because some air that was originally contained in the damping chamber would now need to be withdrawn from the atmosphere. Hence the pressure within the damping chamber would become negative relative to the atmosphere. This negative pressure increases the net driving pressure. Therefore, the FLEX resistance would gradually move from being lower than static model to being higher than static model towards the end of the expiration.

Obviously, the damping effect can play a role in changing the dynamic resistance of the FLEX device. The degree of damping is alterable and was changed during the experiment mainly to smooth out any sudden change in resistance or to damp out any oscillation.

The compensation of tidal volume varied from 20mL to 100mL depending on the compliance of the model lung, the airway resistance, the range of travel (elevation) of the flow-control valve and diaphragm. A stiffer lung and more occluded airway required more compensation. Meanwhile, more travel of the diaphragm increased the dead space within the circuit, hence more compensation was needed.
